# Trends in caesarean section and instrumental deliveries in relation to Body Mass Index: a clinical survey during 1978 - 2001

**DOI:** 10.1186/1742-4755-7-18

**Published:** 2010-07-22

**Authors:** Gunilla Sydsjö, Adam Sydsjö, Jan Brynhildsen, Ann Josefsson

**Affiliations:** 1 Division of Obstetrics and Gynaecology, Department of Clinical and Experimental Medicine, Faculty of Health Sciences, Linköping University, SE-581 85 Linköping, Sweden

## Abstract

**Background:**

During the last 20 years the rate of CS has increased in Sweden as it has in many other countries. The proportion of pregnant women suffering from a high BMI has also increased rapidly during the same time period. It would therefore be of interest to study both how and if these two observations are related to each other. The aim was therefore to study trends in mode of caesarean section (CS) and instrumental deliveries among women in three BMI groups over a time span of almost 25 years with special focus on the observed body weight of pregnant women.

**Method:**

The design is a retrospective cohort study using medical records of consecutively delivered women at two delivery wards in South East Sweden during the years 1978, 1986, 1992, 1997 and 2001.

**Results:**

No significant time-trends were found for CS and instrumental delivery within each BMI-group for the time period studied. The proportion of women with BMI ≥ 25 delivered by means of CS or instrumental delivery increased quite dramatically from 1978 to 2001 (χ^2 ^test for trend; p < 0.001 for both CS and instrumental deliveries). The mean birth weight in relation to BMI and year of study among women delivered by means of CS decreased, a trend that was most evident between 1997 and 2001 (F-test; p = 0.005, p = 0.004, and p = 0.003 for BMI < 20, 20-24.9, and ≥ 25, respectively).

**Conclusion:**

Overweight and obese pregnant women constitute a rapidly growing proportion of the total number of CS and instrumental deliveries. Planning and allocation of health resources must be adjusted to this fact and its implications.

## Background

Swedish women have experienced an almost epidemic increase in body weight in recent decades, as have women in western societies in general [1,2]. In addition to the general health complications that are the same for both sexes, women of fertile age are also more susceptible to reproductive hazards in connection with pregnancy and childbirth [3].Obese women have an increased risk of several complications during pregnancy and delivery [4,5]. There is also an increased risk for neonatal complications [4-6]. Overweight and obese pregnant women are also at increased risk for instrumental deliveries employing, for example, forceps, vacuum extraction or caesarean section (CS), which are procedures not completely free of risk even among women of a normal body constitution [3,5-7]. The risks connected with instrumental deliveries include perineal tears, thromboembolic complications, wound ruptures and infections. Furthermore, a CS will lead to a higher proportion of uterine scars that may cause trouble in the following pregnancy.

During the last 20 years the rate of CS has increased in Sweden as well as in many other countries [8]. The proportion of pregnant women suffering from a high BMI has also increased rapidly during the same time period. It would therefore be of interest to study how and in what ways these two changes might be related to each other. The Swedish Medical birth register maintained by the Board of Health and Welfare covers pregnancy data on each mother's weight only since 1992. To incorporate other parameters of possible importance for delivery outcome, parameters that have not been available in the Swedish birth register until recently, we decided to utilize a manually compiled database on pregnancy and delivery with data from 1978 until 2001 [9]. We hypothesised, that given that the same indications to perform an instrumental delivery or CS were relevant, no substantial increase should be found over time among pregnant women in three different BMI groups.

## Methods

Information from an already available database on women who gave birth at two different Swedish hospitals in 1978, 1986, 1992, 1997 and 2001 was utilized. All information for the years 1978, 1986, 1992 and 1997 was collected manually. Data from 2001 were extracted from the computerized file system (Obstetrix^®^, Siemens). This particular database was originally collected in order to study shifts in sickness absence during pregnancy. These results are published elsewhere [8,9]. The study years were chosen as they represented shifts in the various social security benefits introduced in Sweden over the same time. Swedish antenatal, delivery and neonatal information are registered in standardised and identical records. No major changes of the records occurred during the study period. The following data were collected: the pregnant woman's age, parity, height and weight at the beginning of the pregnancy. Relevant information such as smoking habits and occupation were also extracted. Delivery data such as gestational week at delivery and mode of delivery were retrieved from the delivery records.

If any of the required parameters was missing in the antenatal or delivery records the woman was excluded. Hence, of the original 4 911 delivered women, 3 798 (77.3%) could be included. BMI was calculated on information from the woman's first visit at the antenatal care clinic (ACC), which occurs in gestational week 8-10. Gestational length was calculated by ultrasound for all women, with the exception of 1978, when date of last menstrual period still was used.

### Ethics

The study was approved by the Human Research Ethics Committee, Faculty of Health Sciences, Linköping University.

### Statistics

Differences *between *the three BMI-groups (i.e. < 20, 20-24.9, and ≥ 25) studied with regard to caesarean sections and instrumental deliveries during each year of study (i.e. 1978, 1986, 1992, 1997, and 2001) were tested by means of the χ^2 ^test. In order to be able to adjust for potentially important background characteristics, multiple logistic regression analysis was used. In these analyses, caesarean sections and instrumental deliveries (coded as yes/no) were defined as dependent variables and BMI-group, parity (no previous children/previous children), age, smoking habits (yes/no), occupational status (gainfully employed/not gainfully employed), and twinning (yes/no) were defined as independent variables. We also tested if there were any indications of time-trends for caesarean sections or instrumental deliveries *within *each BMI-group by means of the χ^2 ^test for trend.

## Results

Among the 3 798 women studied, 25.5% had a BMI of 25 or more and 17.6% had a BMI less than 20, and the proportion of overweight women increased by time (χ^2 ^test for trend; p < 0.001) (Table 1). Table 1 further shows that, on average during the time period, 12.5% of the women studied had been delivered by means of CS and that 7.2% of the deliveries were classified as instrumental (forceps or vacuum extraction). The proportion of CS also increased over the time period studied (χ^2 ^test for trend; p < 0.001).

**Table 1:** Characteristics of the study population

	Year of study					Total
	1978	1986	1992	1997	2001	
**No. of studied women**	595 (656)	654 (671)	925 (1178)	604 (863)	1 020 (1543)	3 798 (4911)
**Categorized BMI**						
< 20	186 (31.3%)	153 (23.4%)	152 (16.4%)	75 (12.4%)	103 (10.1%)	669 (17.6%)
20-24.9	343 (57.6%)	416 (63.6%)	542 (58.6%)	334 (55.3%)	526 (51.6%)	2 161 (56.9%)
≥ 25	66 (11.1%)	85 (13.0%)	231 (25.0%)	195 (32.3%)	391 (38.3%)	968 (25.5%)
**No. of Cesarean sections**	54 (9.1%)	74 (11.3%)	118 (12.8%)	58 (9.6%)	171 (16.8%)	475 (12.5%)
**No. of instrumental deliveries**	54 (9.1%)	44 (6.7%)	33 (3.6%)	48 (7.9%)	94 (9.2%)	273 (7.2%)

**Figure 1:**
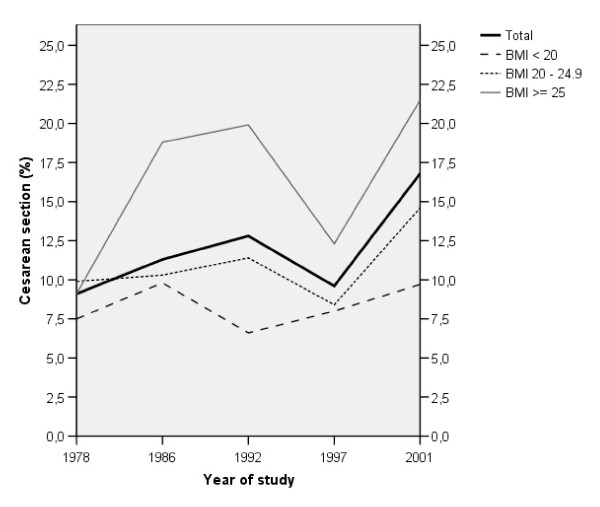
**Percentage of Caesarean sections in relation to BMI and year of study**.

There were differences in the proportion of CS among the women in the different BMI-groups during the years 1992 and 2001 (χ^2 ^test; p < 0.001 and 0.003, respectively), see Figure 1. In 1992, the OR for being delivered by CS was 3.76 (95% CI: 1.82-7.76) among the women whose BMI was ≥ 25, compared to women with BMI < 20 (p < 0.001), even after adjustments for certain background characteristics (i.e. parity, age, smoking habits, occupational status, and twinning). The corresponding OR and CI were 2.78 (1.33-5.82) during the year 2001 (p = 0.007). Sub-analyses was performed among the women who gave birth in 1978, 1986, 1992, and 1997, in which CS were divided into 'acute' and 'elective', respectively (data not shown; women who gave birth in 2001 were excluded as we did not have sufficient information to further categorize them). The only difference found between BMI-groups in these sub-analyses was among women who gave birth in 1992, at which point women who's BMI was ≥ 25 were more likely than others to have an elective CS (χ^2 ^test; p = 0.007). In Figure 1, no significant time-trends for CS within each BMI-group were found for the time period studied (i.e. 1978-2001); the most pronounced indication was found for women whose BMI was ≥ 25 (χ^2 ^test for trend; p = 0.081).

**Figure 2:**
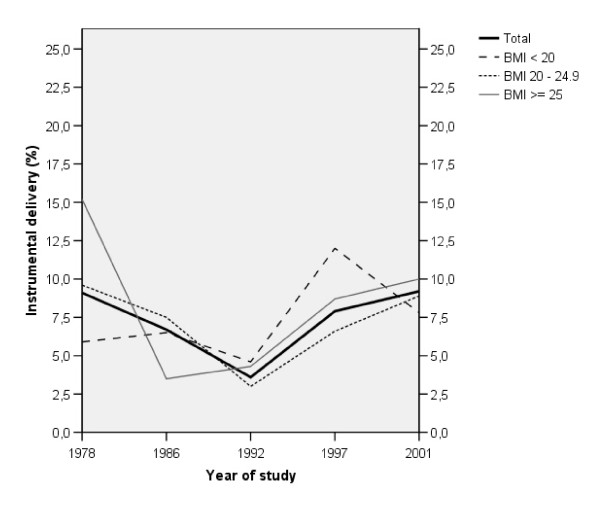
**Percentage of instrumental deliveries in relation to BMI and year of study**.

Figure 2 displays the proportion of instrumental deliveries in relation to BMI-group and year of study. The χ^2 ^tests revealed no differences between BMI-groups during the years, but when adjustments were made for background characteristics there was an overrepresentation of instrumental deliveries among women whose BMI was ≥ 25, compared to those with a BMI < 20, during the year 1978; OR = 4.01 (95% CI: 1.53-10.50), p = 0.005. In Figure 2, no time-trends for instrumental deliveries were evident within each BMI-group between 1978 and 2001. However, when the first study year (i.e. 1978) was excluded from the analyses, a positive trend was found among women with a BMI of ≥ 25 (χ^2 ^test for trend; p = 0.004).

**Figure 3:**
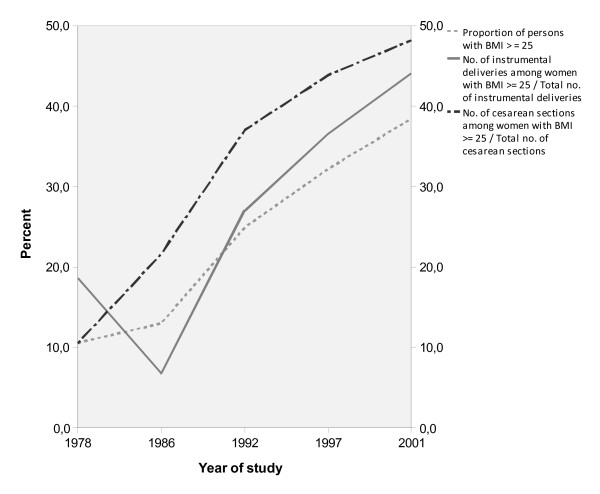
**Percentage of women with BMI ≥ 25 among women delivered by means of Caesarean section or instrumental deliveries during the study period**.

In Figure 3, the percentage of women with BMI ≥ 25 among those delivered by means of CS or instrumental deliveries, respectively, are displayed. The proportion of women with BMI ≥ 25 in both groups increased quite dramatically from 1978 to 2001 (χ^2 ^test for trend; p < 0.001 for both CS and instrumental deliveries).

Table 2 shows the mean birth weight in relation to BMI and year of study among women delivered by means of CS. Differences were found between the three BMI-groups during the years 1978 and 2001 (F-test; p = 0.022 and 0.015, respectively). There were also significant differences over time within each BMI-group concerning birth weight, mainly evident as a decrease between 1997 and 2001 (F-test; p = 0.005, p = 0.004, and p = 0.003 for BMI < 20, 20-24.9, and ≥ 25, respectively).

**Table 2:** Mean birth weight in relation to BMI and year of study among women delivered by means of Caesarean section

	BMI < 20		BMI 20-24.9		BMI > = 25	
	Mean	N	Mean	N	Mean	N
1978	2905	14	332	34	3916	6
1986	3120	15	3047	42	3320	16
1992	3293	9	3407	61	3523	45
1997	3110	6	3533	28	3629	23
2001	1994	10	2981	77	3047	84

5 children's birth weight was uncertain therefore excluded.

## Discussion

In this study we did not find any significant time-trends for CS or instrumental deliveries within each BMI-group from 1978 to 2001. However, from 1986 on, it seems as if women with a BMI of ≥ 25 were delivered with an instrumental delivery to a greater extent than prior to that time.

The increased risk of CS and instrumental deliveries in overweight and obese women is well known [5]. The results from the present study, however, points out that the relative proportion of overweight and obese women in the CS- and instrumental delivery groups has increased. In 1978, 11.1% of all women were overweight or obese and the relative proportion of these women in the CS and instrumental delivery groups were 11% and 18% respectively. In 2001, 38.3% of all women were at least overweight whereas these women constituted 49% and 42% of the CS and instrumental deliveries respectively.

Overall the proportion of women with BMI ≥ 25 delivered by means of CS or instrumental deliveries increased quite dramatically from 1978 to 2001, which probably is explained by the general increase of overweight and obesity in the population [1,2,10]. The temporary decline in CS incidence in 1997 is most probably explained by local intervention programs to break the trend of increasing CS rates. Apparently, these programs did not have any long lasting effects. We also found that the mean birth weight in relation to BMI and year of study among women delivered by means of CS decreased. This decrease was evident mainly after 1997 and might be explained by wider indications, new technologies have been introduced in order to identify foetuses at risk during delivery and an increased readiness to perform a CS over time [11]. Although this clinical study material is rather small, it contains valuable information, which would have been difficult to obtain through national databases or other sources.

It is also a strength of the study that the material was collected through prospectively gathered information in standardised antenatal and delivery records and not by maternal recall. In this study, the BMI is calculated from information on weight and height as measured at the first visit to antenatal care clinic, in gestational week 8-10 week which adds to the credibility of the study. The dropout was evenly distributed for all years of the study and was not considered of importance for the result as it is believed to depend on underreporting in the standardised file. Except from BMI, gestational weight gain has been identified as another aspect which might increase the risk for CS irrespective of BMI [12].

## Conclusion

In conclusion, it is evident that overweight and obese pregnant women constitute a rapidly growing proportion of the total amount of CS and instrumental deliveries. Planning and allocation of health resources must be adjusted to this fact and its implications.

## Competing interests

The authors declare that they have no completing interests.

## Authors' contributions

GS designed the study, data collection, analysis of data, preparation of the manuscript, AS research idea and design, analyses of data, preparation of the manuscript, JB analysis of data, preparation of the manuscript, AJ analysis of data, preparation of the manuscript. All authors read and approved the final manuscript.
